# Impaired Circulating CD4^+^LAP^+^ Regulatory T Cells in Patients with Acute Coronary Syndrome and Its Mechanistic Study

**DOI:** 10.1371/journal.pone.0088775

**Published:** 2014-02-18

**Authors:** Zheng-Feng Zhu, Kai Meng, Yu-Cheng Zhong, Liang Qi, Xiao-Bo Mao, Kun-Wu Yu, Wei Zhang, Peng-Fei Zhu, Ze-Peng Ren, Bang-Wei Wu, Qin-Wei Ji, Xiang Wang, Qiu-Tang Zeng

**Affiliations:** 1 Laboratory of Cardiovascular Immunology, Key Laboratory of Biological Targeted Therapy of the Ministry of Education, Institute of Cardiology, Union Hospital, Tongji Medical College of Huazhong University of Science and Technology, Wuhan, China; 2 Department of Radiology, the First Affiliated Hospital of Nanjing Medical University, Nanjing, China; 3 Department of Cardiology, the People's Hospital of Guangxi Zhuang Autonomous Region, Nanning, China; Virginia Commonwealth University, United States of America

## Abstract

**Objective:**

CD4^+^ latency-associated peptide (LAP)^+^ regulatory T cells (Tregs) are a newly discovered T cell subset in humans and the role of these cells in patients with acute coronary syndrome (ACS) has not been explored. We designed to investigate whether circulating frequency and function of CD4^+^LAP^+^ Tregs are defective in ACS.

**Methods:**

One hundred eleven ACS patients (acute myocardial infarction and unstable angina) and 117 control patients were enrolled in the study. The control patients consisted of chronic stable angina (CSA) and chest pain syndrome (CPS). The frequencies of circulating CD4^+^LAP^+^ Tregs and the expression of the transmembrane protein glycoprotein-A repetitions predominant (GARP) on CD4^+^ T cells were determined by flow cytometry. The function of CD4^+^LAP^+^ Tregs was detected using thymidine uptake. Serum interleukin-10 (IL-10) and transforming growth factor-β protein (TGF-β) levels were detected using ELISA and expression of *GARP* mRNA in peripheral blood mononuclear cells (PBMCs) was measured by real time-polymerase chain reaction.

**Results:**

We found ACS patients had a significantly lower frequency of circulating CD4^+^LAP^+^ Tregs, and the function of these cells was reduced compared to controls. The expression of *GARP* in CD4^+^ T cells and the serum levels of TGF-β in ACS patients were lower than those of control patients. The serum levels of IL-10 were similar between the two cohorts.

**Conclusions:**

A novel regulatory T cell subset, defined as CD4^+^LAP^+^ T cells is defective in ACS patients.

## Introduction

Atherosclerosis is a chronic inflammatory disease involving immunologic imbalance. Various immune cells, macrophagocyte, monocyte, lymphocyte especially T lymphocytes participate in the chronic inflammatory reaction and ultimately lead to the occurrence and development of acute coronary syndrome (ACS) [1]–[4]. Regulatory T cells (Tregs) play an important role in maintaining peripheral tolerance, preventing autoimmune diseases, and restraining chronic inflammatory diseases [5]–[8]. Previous studies have shown that naturally occurring CD4^+^CD25^+^ Tregs are down-regulated in patients with acute coronary syndrome (ACS) [9]–[11].

The classical Treg phenotype is defined as CD4^+^CD25^+^FOXP3^+^ T cells [12]–[13]. Recently, Weiner laboratory identified a novel population of human Tregs in peripheral blood that were characterized by the expression of latency-associated peptide (LAP) [14]. LAP is a linker pro-peptide that is specific for the active form of transforming growth factor-β (TGF-β) [15]–[17]. TGF-β is secreted as a latent complex in which the N-terminal region is non-covalently associated with LAP, while the C-terminal homodimer corresponds to mature TGF­β. In order for latent TGF-β to become active, the mature TGF- β must be released from LAP [16]–[18]. The CD4^+^LAP^+^ T cells lack Foxp3 expression, but they function similarly to the classical CD4^+^CD25^+^FOXP3^+^ Tregs and produce a suppressive effect on immune response. *In vitro*, the suppressive activity of CD4^+^LAP^+^ T cells is dependent on TGF-β, interleukin-10 (IL-10), and cell-cell contact [14]. CD4^+^LAP^+^ T cells have been shown to suppress murine autoimmunity in experimental models of encephalomyelitis, systemic lupus erythematosus, colitis, and diabetes [24]–[30]. Glycoprotein A repetitions predominant (GARP), an 80-kDa type I transmembrane glycoprotein leucine rich repeat (LRR), is highly expressed in activated Tregs [19]–[21]. GARP binds directly to LAP and tethers latent TGF­β on the surface of activated Tregs, and it has been clarified that GARP is a receptor for latent TGF­β [22]–[23].

The role of this novel subset of Tregs has been studied in many diseases, including systemic lupus erythematosus, experimental autoimmune encephalomyelitis, diabetes, and atherosclerosis [24]–[31]. The status of CD4^+^LAP^+^ Tregs in patients with ACS has not yet been defined, however. Here we compared the circulating frequency of CD4^+^LAP^+^ Tregs in ACS patients with the frequency in control patients (chronic stable angina, CSA and chest pain syndrome, CPS). Levels of CD4^+^LAP^+^ Tregs were down regulated and their function was reduced in ACS patients. The LAP receptor GARP expression on CD4^+^ T cells from patients with ACS was also reduced.

## Materials and Methods

### Patients

Two hundred twenty-eight patients from Wuhan Union Hospital were classified into three groups: (1) acute coronary syndrome (ACS) group(including acute myocardial infarction (AMI) and unstable angina (UA), 111 patients in total; 64 men and 47 women; mean age, 59±7 years; inclusion criteria: acute myocardial infarction confirmed by significant rise of creatine kinase-MB and troponin I levels and/or not ST segment elevation, and unstable angina confirmed by chest pain at rest with definite ischemic proof, including ST-segment changes and/or T-wave inversion and angiographic evidence of coronary artery stenosis (>70%)); (2) chronic stable angina (CSA) group (29 men and 21 women, mean age 58±9, inclusion criteria: effort angina (lasting >3 months and without a previous history of unstable angina or myocardial infarction and angiographic evidence of coronary artery stenosis (>70%)); and (3) the chest pain syndrome (CPS) group (35 men and 32 women, mean age 57±6, inclusion criteria: chest pain not accompanied by electrocardiographic changes, coronary artery stenosis (Coronary angiography or coronary CTA), or coronary spasm [32]). The exclusion criteria included the following: (1) patients treated with anti-inflammatory drugs such as non-steroidal anti-inflammatory drugs and steroids; (2) those who have diseases including connective tissue disease, thromboembolism, disseminated intravascular coagulation, advanced liver disease, renal failure, malignant disease, or other inflammatory disease (such as septicemia or pneumonia); (3) those who have other heart disease such as rheumatic heart disease, valvular heart disease or congenital heart disease, or atrial fibrillation or those using a pacemaker.

### Ethics statement

The investigation conforms to the principles outlined in the Declaration of Helsinki. The trial was approved by the ethics committee of Tongji Medical College of Huazhong University of Science and Technology. Patients and controls provided written informed consent.

### Sample preparation and peripheral blood mononuclear cell (PBMC) isolation

Blood samples were obtained from all the patients within 24 hours after admission. PBMCs were isolated by Ficoll density gradient centrifugation and were used for flow cytometric analysis and FACS sort and cell culture and real time-polymerase chain reaction (RT-PCR). Serum was collected after centrifugation, aliquoted, and frozen at −80°C for subsequent determination of cytokine expression.

### Flow cytometric analyses

PBMCs were stained with anti-human CD4-FITC (R&D Systems) and anti-human LAP-PE (clone 27232, R&D Systems) for 30 min at 4°C. Anti-mouse IgG1-PE (R&D Systems) isotype controls were used to enable normalization and confirm antibody specificity. Antibodies were used without dilution. Stained cells were analyzed by flow cytometry using a FACS Calibur machine (BD).

PBMCs were resuspended at a density of 2×10^6^ cells/ml in RPMI 1640 (ATCC modification A1049101 Gibco) supplemented with 10% heat-inactivated fetal calf serum(Gibco), 10% non-essential amino acids solution (Gibco), 100 U/ml penicillin, and 100 U/ml streptomycin. The cell suspension was seeded in 24-well culture plates. Cells were stimulated by exposure to soluble anti-CD3 (eBioscience, 5 µg/ml) and anti-CD28 (eBioscience, 2 µg/ml each) for 24 hours. The incubator was set at 37°C under a 5% CO_2_ environment. After 24 hours, the cells were harvested and stained with anti-human LAP-APC (clone 27232, R&D Systems), anti-human CD4-FITC (R&D Systems), and anti-human GARP-PE (G14D9, eBioscience) for 30 min at 4°C. Antibodies were used without dilution. Following the surface staining, cells were analyzed by flow cytometry with FACS Calibur (BD).

### Proliferation and suppression assays

**Figure 1:**
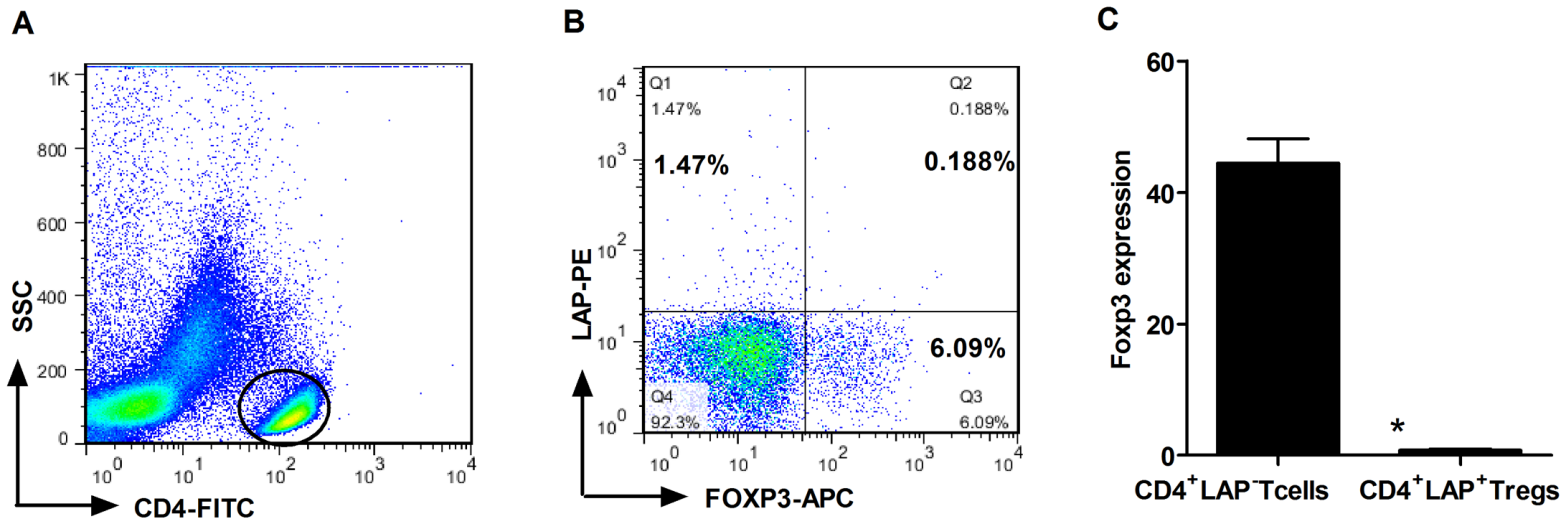
Foxp3 was not expressed on CD4^+^LAP^+^ Tregs. (A) Representative FSC/SSC dot plot shows the gated CD4^+^ T cells. (B) Freshly isolated PBMCs were stained with CD4-FITC, LAP-PE, Foxp3-APC. We found no co-expression of LAP and Foxp3 on CD4 T cells. (C) RT-PCR analysis to determine Foxp3 expression in respective FACS-sorted populations.

PBMCs were stained with anti-human CD4-FITC (R&D Systems), anti- human-LAP-PE (R&D Systems), and anti-human CD25-PerCP (Biolegend) for 30 min at 4°C. Antibodies were used without dilution. After the surface staining, the responder T cells (Tresps; CD4^+^LAP^−^CD25^int/low^ T cells) and CD4^+^LAP^+^ Tregs were obtained by FACS sorting using a FACs Aria (BDBiosciences). The purity of CD4^+^LAP^−^CD25^int/low^ T cells was >97%, and the purity of CD4^+^LAP^+^ Tregs was >95%. In order to make a distinction between CD4^+^LAP^+^ Tregs and CD4^+^CD25^+^FOXP3^+^ Tregs, we confirmed that there was no Foxp 3 expression on the subset of CD4^+^LAP^+^ Tregs using flow cytometry and RT-PCR (Figure 1). Next we designed three experiments to investigate the function of CD4^+^LAP^+^ Tregs: 1) CD4^+^LAP^−^CD25^int/low^ Tresps cultured alone, 2)CD4^+^LAP^−^CD25^int/low^ Tresps and CD4^+^LAP^+^ Tregs were co-cultured at different ratios (Tregs/Tresps ratios: 1∶1, 1∶2 and 1∶4, 3) crossover experiment; CD4^+^LAP^+^ Tregs from ACS patients were co-cultured with CD4^+^LAP^−^CD25^int/low^ Tresps from controls (CPS and CSA patients) at a 1∶1 ratio, CD4^+^LAP^+^ Tregs from controls were co-cultured with CD4^+^LAP^−^CD25^int/low^ Tresps from ACS patients at a 1∶1 ratio. T cells were incubated in complete RPMI 1640 (ATCC modification A1049101 Gibco) supplemented with 10% heat-inactivated fetal calf serum (Gibco), 10% non-essential amino acids solution (Gibco), 100 U/ml penicillin, and 100 U/ml streptomycin at 37°C with plate-bound (5 µg/ml, eBioscience) anti-CD3 and soluble anti-CD28 (2 µg/ml, eBioscience) in 5% CO_2_ for 72 h in U-bottom 96-well plates. Antibodies were used without dilution. All cells were cultured in a final volume of 200 µl. [^3^H]-thymidine (1 µl, Amersham Biosciences) was added to each well 16 h before the harvest of cells and the incorporation of [^3^H]-thymidine was assayed by scintillation counting (PerkinElmer).

### Real-time PCR

RNA was extracted from freshly isolated PBMCs and CD3/28-sitmulated PBMCs (as described above) with RNAiso Plus (Takara Biotechnology) and reverse transcribed to cDNA using a RNA PCR Kit (Takara), according to the manufacturer's instructions. Expression of target genes was quantified using the SYBR Green Master Mix (Takara) on an ABI Prism 7900 Sequence Detection system (Applied Biosystems). All reactions were performed in at least duplicate for each sample. Primer pairs were as follows:

GARP: Forward: 5′-CCCTGTAAGATGGTGGACAAGAA-3′;

Reverse: 5′-CAGATAGATCAAGGGTCTCAGTGTCT-3′

β-actin: Forward:5′-TCGTCCACCGCAAATGCTTCTAG-3′

Reverse: 5′-ACTGCTGTCACCTTCACCGTTCC-3′

Relative mRNA expression levels were calculated using the comparative CT methodformula 2-^ΔΔCT^. Data were normalized to β-actin [33].

### Cytokines detection

The plasma levels of TGF-β1 and IL-10 were measured by enzyme-linked immunosorbent assay (ELISA) according to the manufacturer's instructions (both from R&D Systems). The detection limits were 4.61 pg/mL for TGF-β1 and 3.9 pg/ml for IL-10. The intra-assay and inter-assay variation coefficients for all ELISA were <10%. All samples were measured in duplicate.

### Statistical analyses

Data are expressed as the mean±SEM in the figures. Differences were evaluated using one-way ANOVA for multiple comparisons, followed by a post hoc Student-Newmann-Keuls test and multivariate analysis when necessary. For the ranked data, Pearson's X^2^ test or Fisher's exact test was performed for the comparison between groups. All analyses were conducted using SPSS (Statistical Package for the Social Sciences) 17.0 software, and statistical significance was set at p<0.05.

## Results

Basic clinical characteristics of the study population are summarized in Table 1. There were no significant differences in age, gender, diabetes mellitus status, hypercholesterolemic status, or the use of aspirin or calcium blockers, or smoking status among the patients with ACS, CSA, or CPS groups. There were significant differences in hypertension and the use of clopidogrel, β-blockers, statins, angiotensin-converting enzyme inhibitors (ACEIs), angiotensin receptor blockers (ARBs), and nitrates among the ACS, CSA, and CPS groups. However, we have compared CD4^+^LAP^+^ Tregs levels between patients with and without ongoing treatment with clopidogrel, β—blockers, statins, angiotensin-converting enzyme inhibitors/angiotensin receptor blockers, or nitrate, and these factors did not influence CD4^+^LAP^+^ Tregs expression use multivariate analysis, moreover, as shown previously, the circulating CD4^+^LAP^+^ Tregs levels were also not correlated with hypertension (data not shown) [48].

**Table 1:** Clinical characteristics of the study population.

Characteristics	CPS (n = 67)	CSA(n = 50)	ACS(n = 111)	P
Age(years)	57±6	58±9	59±7	P = 0.21
Sex(male/female)	35/32	29/21	64/47	P = 0.67
Risk factors (n (%))				
Hypertension	18(26.9%)	22(44%)	53(47.7%)	P = 0.020
Diabetes	19(28.4%)	19(38%)	44(39.6%)	P = 0.298
Hyperlipidaemia	18(26.9%)	21(42%)	48(43.2%)	P = 0.076
Tobacco	24(35.8%)	26(52%)	58(52.6%)	P = 0.079
Medications (n (%))				
Aspirin	49(73.1%)	41(82%)	94(83.9%)	P = 0.277
Clopidogrel	35(52.2%)	31(62%)	81(73%)	P = 0.018
ACEI/ARBs	23(34.3%)	30(60%)	75(67.6%)	P<0.001
Beta-blockers	21(31.3%)	29(58%)	64(57.7%)	P = 0.001
Calcium blockers	16(23.8%)	17(34%)	35(31.5%)	P = 0.427
Nitrates	11(16.4%)	14(28%)	64(57.78%)	P<0.001
Statins	15(22.4%)	37(74%)	88(79.3%)	P<0.001

Data are presented as mean ± S.D., percentages, or numbers. ACS: acute coronary syndrome, CPS: chest pain syndrome, CSA: chronic stable angina, ACEI: angiotensin-converting enzyme inhibitor, ARB: angiotensin receptor blocker.

### The frequency of circulating CD4^+^LAP^+^ Tregs is decreased in patients with ACS

**Figure 2:**
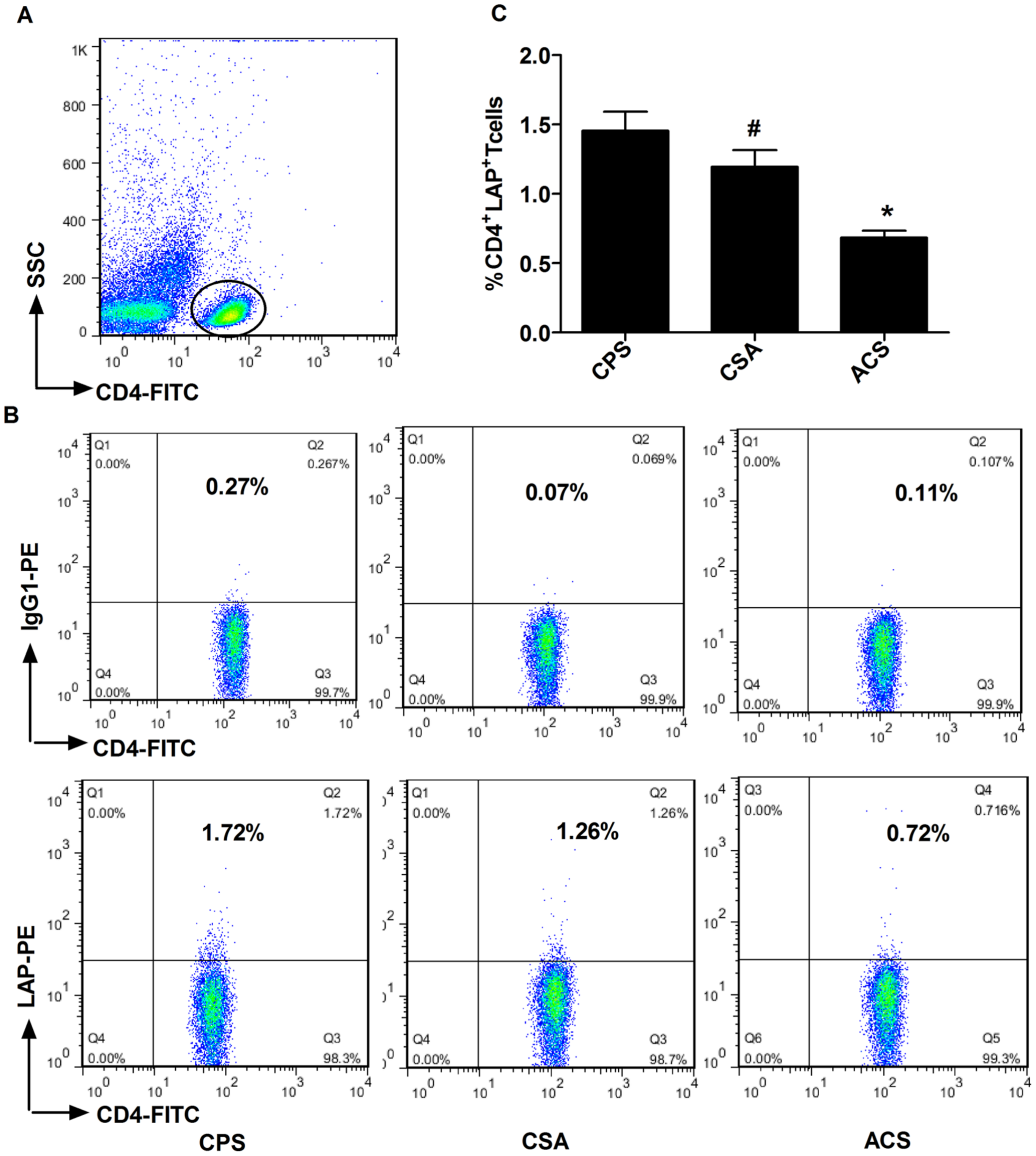
Frequencies of CD4^+^LAP^+^ Tregs in freshly isolated PBMCs from patients with CPS (n = 32), CSA (n = 18), and ACS (n = 44,AMI (n = 16) and UA (n = 28)). (A) Representative FSC/SSC dot plot shows the gated CD4^+^ T cells. (B) Representative FACS analyses of a sample from each group show the frequencies of CD4^+^LAP^+^ Tregs (upper panels show isotype controls). (C) Percentages of CD4^+^LAP^+^ Tregs based on FACs analyses were comparable among ACS, CSA, and CPS groups. * p<0.01 vs. CSA or CPS; # p>0.05 vs. CPS.

We determined the frequency of circulating CD4^+^LAP^+^ Tregs using flow cytometry. We found that the percentage of circulating CD4^+^LAP^+^ Tregs in the CD4^+^ T cell population was decreased significantly in patients with ACS (0.68±0.04%) compared to patients with CSA (1.2±0.12%) or CPS (1.45±0.14%) (*p*<0.001) as shown in Figure 2. There was no obvious difference between the CSA and CPS groups (*p* = 0.21).

### The frequency of circulating CD4^+^GARP^+^ T cells and CD4^+^LAP^+^GARP^+^ T cells is decreased in patients with ACS

**Figure 3:**
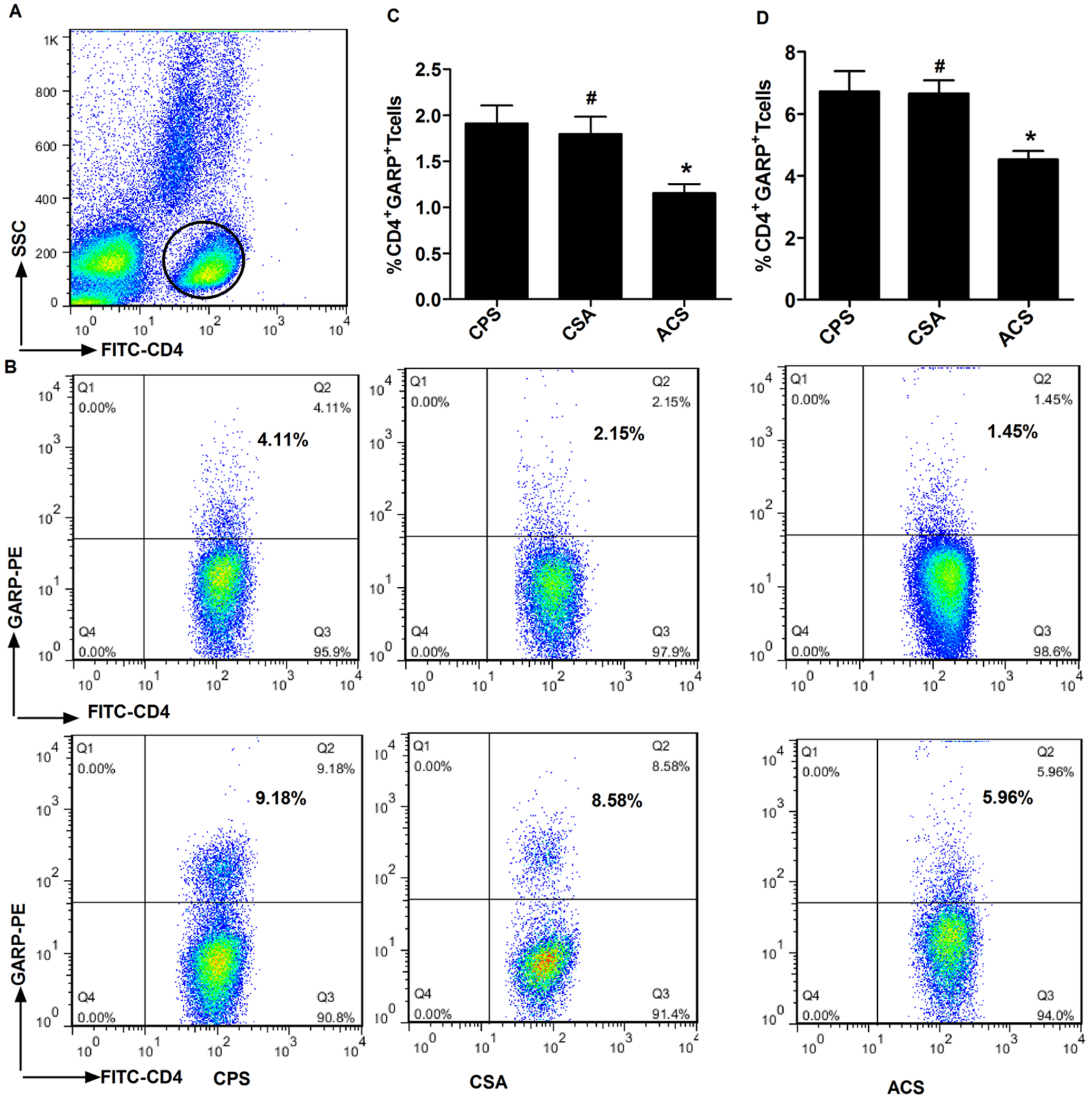
The expression of GARP on CD4^+^ T cells in freshly isolated PBMCs and CD3/28-sitmulated PBMCs. Bood samples were collected from patients with CPS (n = 20), CSA (n = 17) and ACS (n = 37, AMI (n = 18), and UA (n = 19)). PBMCs were freshly isolated or stimulated with CD3/CD28 for 24 h. Then the cells were stained with anti-human CD4-FITC, anti-human GARP-PE and analyzed by flow cytometry using FACS Calibur (BD). (A) Representative dot plot shows the gated CD4^+^ T cells on the FSC/SSC. (B) Representative FACS images show GARP expression on CD4^+^ T cells in unstimulated PMBCs (upper panel) and stimulated PBMCs (lower panel) from one patient in each group. Comparison of the CD4^+^GARP^+^ T cells frequencies in unstimulated PBMCs (C) and stimulated PBMCs (D) among four groups. * *p*<0.01 vs. CSA or CPS; # *p*>0.05 vs. CPS.

GARP is reportedly a receptor of LAP. We therefore measured the expression of GARP on CD4^+^ T cells. As GARP is highly expressed on activated T cells, we first stimulated PBMCs with anti-CD3/28 for 24 hours prior to the assay. GARP expression was also measured on CD4^+^ T cells in freshly isolated PBMCs. Figure 3 shows expression of GARP on CD4^+^ T cells from each patient in the study. In both stimulated and unstimulated PBMCs, the frequencies of CD4^+^GARP^+^ T cells were reduced in ACS patients compared with CSA and CPS patients. In the unstimulated condition, the frequency of circulating CD4^+^GARP^+^ T cells (CD4^+^GARP^+^ T cells/CD4^+^ T cells) was reduced in patients with ACS (1.15±0.10%) compared with those with CSA (1.8±0.18%) or CPS (1.91±0.19%) (*p*<0.005), there was no obvious difference between the CSA and CPS groups (*p* = 0.68). In the stimulated condition, the frequency of CD4^+^GARP^+^ T cells was also reduced in patients with ACS (4.53±0.27%) compared with CSA (6.67±0.42%) and CPS (6.73±0.65%) (*p*<0.005). No obvious difference was found between the CSA and CPS group (*p* = 0.94).

**Figure 4:**
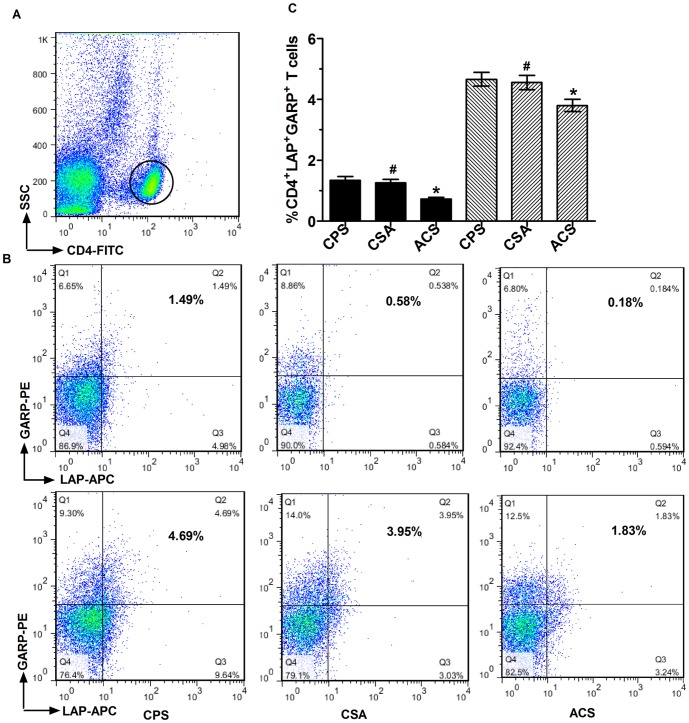
The expression of GARP and LAP on CD4^+^ T cells in freshly isolated PBMCs and CD3/28-sitmulated PBMCs. Bood samples were collected from patients with ACS (AMI (n = 10) and UA (n = 10)) and controls (CSA (n = 17), CPS (n = 20), and PBMCs were freshly isolated or stimulted with CD3/CD28 for 24 h, then the cells were stained with anti-human CD4-FITC,anti-human LAP-APC, anti-human GARP-PE and analyzed the data by flow cytometry using FACS Calibur (BD).(A) Representative dot plot shows the gated CD4 T cells on the FSC/SSC. (B) Representative FACS pictures show the GARP and LAP expreesion on CD4^+^ T cells in unstimulated PMBCs (upper panel) and stimulated PBMCs (lower panel) from one patient in each group. (C) Statistical analysis of the percentage CD4^+^LAP^+^ GARP^+^T cells. Dark bars indicate data from unstimulated samples. Hatched bars indicate data from stimulated samples. *p<0.05 vs. CSA or CPS; # *p*>0.05 vs.CPS.

Next, we evaluated whether levels of LAP and GARP were reduced concomitantly on CD4^+^ T cells from ACS patients (Figure 4). PBMCs were stimulated with anti-CD3/28 and stained with anti-human LAP-APC (R&D Systems), anti-human CD4-FITC, and anti-human GARP-PE for 30 min at 4°C. We also evaluated unstimulated PBMCs. In the unstimulated group, the frequency of CD4^+^LAP^+^GARP^+^ T cells (CD4^+^LAP^+^GARP^+^ T cells/CD4^+^ T cells) was decreased in ACS patients (0.72±0.06%) compared with CSA (1.25±0.11%) and CPS (1.34±0.12%) patients (*p*<0.01), there was no obvious difference between the CSA and CPS groups (*p* = 0.64). In the stimulated group, the percentage of CD4^+^LAP^+^GARP^+^ T cells was also reduced in patients with ACS (3.75±0.21%) compared with CSA (4.55±0.24%) and CPS (4.66±0.24%) (*p*<0.01). Again, no differences were observed between the CSA and CPS groups (*p* = 0.75).

### The function of CD4^+^LAP^+^ Tregs is compromised in patients with ACS

**Figure 5:**
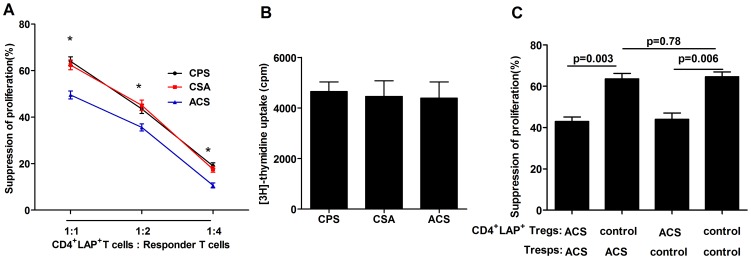
CD4^+^LAP^+^ Tregs from patients with AMI and UA had reduced capacity to suppress responder T cell proliferation. CD4^+^LAP^+^ Tregs and Tresps (CD4^+^LAP^−^CD25^int/low^ T cells) from ACS patients (4 patients from the AMI group and 4 from the UA group), and controls (4 patients from CSA group and 4 patients from CPS) were purified by FACS sorting. (A) Reduced suppressive function of CD4^+^LAP^+^ Tregs from patients with ACS suggested by suppression assay. (B) Similar proliferative capacity of CD4^+^LAP^−^CD25^int/low^ Tresps between ACS group and control (CSA and CPS) group. (C) Crossover experiment with ACS (AMI and UA) and control (CPS and CSA) groups. *p <0.05 vs. Controls.

The ability of CD4^+^LAP^+^ Tregs to inhibit the proliferation of CD4^+^LAP^−^CD25^int/low^ Tresps was determined using a [^3^H]-thymidine incorporation assay in co-cultures of Tregs and Tresps in different ratios (1∶1, 1∶2 and 1∶4). Our data demonstrated that CD4^+^LAP^+^ Tregs from ACS patients exhibited reduced capacity to suppress the proliferation of Tresps at all ratios compared with those from CSA and CPS groups. We next assessed the proliferation of CD4^+^LAP^−^CD25^int/low^ T cells activated by anti-CD3/28; no significant difference was found among the three groups. A crossover experiment between ACS (AMI and UA) patients and control (CPS and CSA) groups also demonstrated that the function of CD4^+^LAP^+^ Tregs from ACS patients was impaired (Figure 5).

### GARP mRNA expression decreased in patients with ACS

**Figure 6:**
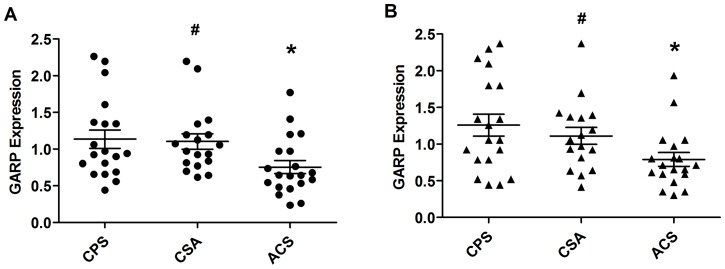
*GARP* mRNA expression in PBMCs of patients with CPS (n = 19), CSA (n = 18), and ACS (n = 20; 8 AMI, 12 UA). The ratio of *GARP* mRNA to *β-actin* mRNA was comparable in stimulated samples (A) and unstimulated samples (B) among the three groups. * *p*<0.05 vs. CSA or CPS; # *p*>0.05 vs. CPS.

We measured the expression *GARP* mRNA by RT-PCR with freshly isolated PBMCs and CD3/28-sitmulated PBMCs. In stimulated PBMCs, the expression of *GARP* was decreased in ACS patients (0.69±0.09) compared with CSA (1.1±0.13) and CPS (1.13±0.13) groups (*p<0.05; Figure 6A). In unstimulated PBMCs, the expression of *GARP* was also decreased significantly in ACS patients (0.75±0.10) compared with CSA (1.12±0.11) and CPS (1.23±0.15) groups (*p<0.05) (Figure 6B). There was no difference in *GARP* expression in cells from CPS and CSA patients.

### Serum levels of TGF-β1decreased in patients with ACS

**Figure 7:**
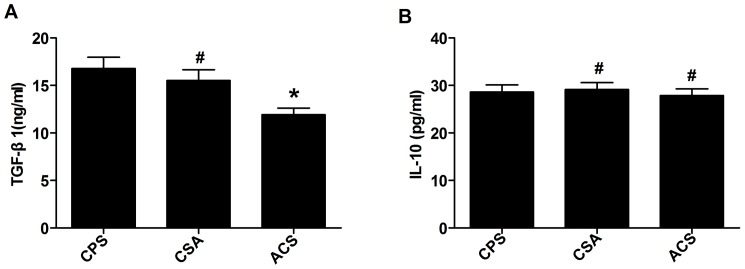
Serum levels of TGF-β and IL-10 in patients with CPS (n = 21), CSA (n = 16), ACS (n = 28; 13 AMI, and 15 UA). (A) The levels of TGF-β were reduced in ACS patient serum compared with that in CSA and CPS patients.* *p*<0.05 vs. CSA or CPS; # *p*>0.05 vs. CPS. (B) The levels of IL-10 were similar among all groups.

TGF-β1 and IL-10 are the main effector cytokines of not only classic Tregs, but also CD4^+^LAP^+^ Tregs [14], [36]. As shown in Figure 7, TGF-β1 levels were reduced in patients with ACS (11.93±0.67 ng/ml) compared with CSA (15.5±1.15 ng/ml) and CPS (16.78±1.19 ng/ml) patients (p<0.05). There was no difference between CPS and CSA patients (p = 0.45). In contrast, there was no difference in IL-10 levels among the three groups (ACS, 27.39±1.37 pg/ml; CSA, 29.3±1.44 pg/ml; and CPS, 28.75±1.51 pg/ml) (p>0.05).

## Discussion

Due to the observation that regulatory T cells are associated with suppression of inflammation, many studies have attempted to elucidate the role of Tregs in inflammatory diseases [35]–[39]. Previous animal studies have demonstrated that enhancements in the numbers of circulating Tregs can alleviate the progression and severity of encephalomyelitis, systemic lupus erythematosus, colitis, diabetes, and heart failure [40]–[43]. Consistent with this purported function, it has been previously shown that acute coronary syndrome (ACS), a chronic inflammatory disease [1]–[4], is associated with a reduction in the number and function of circulating “classical” CD4^+^CD25^+^FOXP3^+^ Tregs [9]–[11], [44]–[46]. Since inflammation is known to be a significant contributor to the pathogenesis of cardiovascular disease, we studied the number and function of a novel subset of regulatory T cells, termed CD4^+^LAP^+^ Tregs, in patients with ACS. This subset of Tregs is stimulated by production of TGF-β and IL-10 and is known to possess potent anti-inflammatory activity (14). We hypothesized that the number and function of CD4^+^LAP^+^ Tregs would be reduced in patients with ACS compared to control patients (CSA and CPS).

We isolated CD4^+^LAP^+^ Tregs from PBMCs harvested from patients with AMI and UA (the ACS groups), and compared the number of Tregs to those of patients with CSA or CPS (control groups). The frequency of circulating CD4^+^LAP^+^ Tregs was significantly reduced in patients with ACS compared to frequencies in patients in the control groups. Furthermore, CD4^+^LAP^+^ Tregs functions of suppressing responder T cells from ACS patients were compromised as demonstrated by analyses of proliferation. These findings are similar to those reported by Cheng *et al.* [9]–[11] and other researchers [44]–[46].

Recent studies suggest that membrane glycoprotein A repetitions predominant (GARP or LRRC32) is a receptor for LAP on the surface of activated human Tregs[22]–[23]. This provided the rationale for us to examine whether the expression of GARP was altered. As expected, we found that the expression of GARP on CD4^+^ T cells was down-regulated in patients with ACS. We also found that percentages of CD4^+^LAP^+^GARP^+^ T cells were reduced in ACS patients compared with controls. GARP can regulate the bioavailability and activation of TGF-β by directly combined with LAP [22], [23], [47]. Thus, we conclude that GARP down-regulation is correlated with the defection of CD4^+^LAP^+^ Tregs. Additional studies are warranted to further evaluate the role of GARP in the pathogenesis of ACS.

Like CD4^+^CD25^+^FOXP3^+^ Tregs, the suppressive activity of CD4^+^LAP^+^ Tregs has been shown to be dependent on both TGF-β1 and IL-10 *in vitro* [14], [34]. We found that serum TGF-β1 levels were reduced in ACS patients compared with those of CSA and CPS patients, but we failed to detect a significant difference in IL-10 levels. Since, CD4^+^LAP^+^ Tregs secrete an array of cytokines, including IL-8, IL-9, IL-10, IFN- γ, and TGF-β [14], the finding that TGF-β1 levels are reduced may suggest a decrease in function and number of circulating CD4^+^LAP^+^ Tregs in patients with ACS. Previous studies have shown that TGF-β can induce surface LAP expression on murine CD4^+^ T Cells[48], therefore, reduced TGF-β1 levels may account for the lower LAP expression on CD4^+^ T cells, and lead to the down-regulated frequency of CD4^+^LAP^+^ Tregs in patients with ACS. Since ACS patients feature higher systemic levels of inflammation, including CRP, TNF-α, IL-6, but lower levels of TGF-β1 [49], CD4^+^LAP^+^ Tregs may be involved in the progression of ACS.

In conclusion, this study is the first to demonstrate that the frequency of circulating CD4^+^LAP^+^ Tregs is reduced and their suppressive function compromised in patients with ACS. A possible mechanism for this effect may be related to the down-regulation of GARP and lower levels of TGF-β1. Yamamoto group demonstrated that local injection of LAP inhibits dermal sclerosis in bleomycin-induced murine scleroderma [50], Sandra Boswell team discovered a new peptide GPC81-95 can induce CD4^+^ T cells surface expression of LAP increase immune suppression of CD4^+^LAP^+^ Tregs [51], suggesting that increasing the numbers and enhancing the function of CD4^+^LAP^+^ Tregs may be a feasible therapeutic approach for treatment of chronic inflammatory diseases like ACS. As CD4^+^LAP^+^ Treg function appears to be compromised in patients with ACS, ACS patients might benefit from this therapeutic strategy. Further studies are warranted to elucidate how to up-regulate the GARP and LAP, thereby raising the frequency of circulating CD4^+^LAP^+^ Tregs in ACS patients. These additional studies are required to comprehensively explore the therapeutic potential of this novel subset of regulatory T cells in coronary heart disease.

## References

[1] Libby P (2002) Inflammation in atherosclerosis. Nature. 420: 868–74. 10.1038/nature01323 12490960

[2] Hansson GK (2005) Inflammation, atherosclerosis, and coronary artery disease. N Engl J Med 352: 1685–95. 15843671 10.1056/NEJMra043430

[3] Hansson GK, Libby P, Schönbeck U, Yan ZQ (2002) Innate and adaptive immunity in the pathogenesis of atherosclerosis. Circ Res 91: 281–91. 12193460 10.1161/01.res.0000029784.15893.10

[4] Libby P (2012) Inflammation in atherosclerosis. Arterioscler Thromb Vasc Biol 32: , 2045–51 10.1161/ATVBAHA.108.179705 PMC3422754 22895665

[5] Shevach EM (2000) Regulatory T cells in autoimmmunity. Annu Rev Immunol 18: 423–49. 10837065 10.1146/annurev.immunol.18.1.423

[6] Sakaguchi S, Yamaguchi T, Nomura T, Ono M (2008) Regulatory T cells and immune tolerance. Cell 33: 775–87. 10.1016/j.cell.2008.05.009 18510923

[7] Vignali DA, Collison LW, Workman CJ (2008) How regulatory T cells work. Nat Rev Immunol 8: 523–32. 18566595 10.1038/nri2343 PMC2665249

[8] Miyara M, Sakaguchi S (2007) Natural regulatory T cells: mechanisms of suppression. Trends MolMed 13: 108–16. 10.1016/j.molmed.2007.01.003 17257897

[9] Cheng X, Yu X, Ding YJ, Fu QQ, Xie JJ, et al (2008) The Th17/Treg imbalance in patients with acutecoronary syndrome. Clin Immunol 127: 89–97. 18294918 10.1016/j.clim.2008.01.009

[10] Mor A, Luboshits G, Planer D, Keren G, George J (2006) Altered status of CD4(+)CD25(+) regulatory T cells in patients with acute coronary syndromes. Eur Heart J 27: 2530–7. 16954132 10.1093/eurheartj/ehl222

[11] Sardella G, De Luca L, Francavilla V, Accapezzato D, Mancone M, et al (2007) Frequency of naturally-occurring regulatory T cells is reduced in patients with ST-segment elevation myocardial infarction. ThrombRes 120: 631–634. 10.1016/j.thromres.2006.12.005 17261328

[12] Hori S, Nomura T, Sakaguchi S (2003) Control of regulatory T cell development by the transcription factor Foxp3. Science 299: 1057–61. 12522256 10.1126/science.1079490

[13] Sakaguchi S, Ono M, Setoguchi R, Yagi H, Hori S, et al (2006) Foxp3+ CD25+ CD4+ natural Regulatory T cells in dominant self-tolerance and autoimmune disease. Immunol Rev 212: 8–27. 16903903 10.1111/j.0105-2896.2006.00427.x

[14] Gandhi R, Farez MF, Wang Y, Kozoriz D, Quintana FJ, et al (2010) Cutting edge: human latency-associated peptide+ T cells: a novel regulatory T cell subset. J Immunol184: 4620–4. 10.4049/jimmunol.0903329 PMC2904991 20368276

[15] Massagué J, Blain SW, Lo RS (2000) TGF-beta signaling in growth control, cancer, and heritable disorders. Cell 103: 295–309. 11057902 10.1016/s0092-8674(00)00121-5

[16] Blobe GC, Schiemann WP, Lodish HF (2000) Role of transforming growth factor beta in human disease. N Engl J Med 342: 1350–8. 10793168 10.1056/NEJM200005043421807

[17] Annes JP, Munger JS, Rifkin DB (2003) Making sense of latent TGF-beta activation. J Cell Sci 116: 217–224. 12482908 10.1242/jcs.00229

[18] Keski-Oja J, Koli K, von Melchner H (2004) TGF-beta activation by traction? Trends Cell Biol 14: 657–9. 15564041 10.1016/j.tcb.2004.10.003

[19] Wang R, Wan Q, Kozhaya L, Fujii H, Unutmaz D (2008) Identification of a regulatory T cell specific cell surface molecule that mediates suppressive signals and induces Foxp3 expression. PLoS One 3: e2705. 18628982 10.1371/journal.pone.0002705 PMC2442191

[20] Battaglia M, Roncarolo MG (2009) The Tregs' world according to GARP. Eur J Immunol 39: 3296–300. 19904770 10.1002/eji.200940117

[21] Ollendorff V, Noguchi T, deLapeyriere O, Birnbaum D (1994) The GARP gene encodes a new member of the family of leucine-rich repeat-containing proteins. Cell Growth Differ 5: 213–9. 8180135

[22] Tran DQ, Andersson J, Wang R, Ramsey H, Unutmaz D, et al (2009) GARP (LRRC32) is essential for the surface expression of latent TGF-beta on platelets and activated FOXP3+regulatory T cells. Proc Natl Acad Sci U S A 106: 13445–50. 19651619 10.1073/pnas.0901944106 PMC2726354

[23] Stockis J, Colau D, Coulie PG, Lucas S (2009) Membrane protein GARP is a receptor for latent TGF-beta on the surface of activated human Treg. Eur J Immunol 39: 3315–22. 19750484 10.1002/eji.200939684

[24] Wu HY Center EM, Tsokos GC, Weiner HL (2009) Suppression of murine SLE by oral anti-CD3: inducible CD4+CD25-LAP+ regulatory T cells control the expansion of IL-17+ follicular helper T cells. Lupus 18: 586–96. 19433458 10.1177/0961203308100511 PMC2753460

[25] Ochi H, Abraham M, Ishikawa H, Frenkel D, Yang K, et al (2006) Oral CD3-specific antibody suppresses autoimmune encephalomyelitis by inducing CD4+CD25–LAP+T cells. Nat Med 12: 627–35. 16715091 10.1038/nm1408

[26] Oida T, Zhang X, Goto M, Hachimura S, Totsuka M, et al (2003) CD4+CD25- T cells that express latency-associated peptide on the surface suppress CD4+ CD45RBhigh-induced colitis by a TGF-beta-dependent mechanism. J Immunol 170: 2516–22. 12594277 10.4049/jimmunol.170.5.2516

[27] Ishikawa H, Ochi H, Chen ML, Frenkel D, Maron R, et al (2007) Inhibition of autoimmune diabetes by oral administration of anti-CD3 monoclonal antibody. Diabetes 56: 2103–9. 17456848 10.2337/db06-1632

[28] Wu HY, Quintana FJ, Weiner HL (2008) Nasal anti-CD3 antibody ameliorates lupus by inducing an IL-10-secreting CD4+ CD25- LAP+ regulatory T cell and is associated with down-regulation of IL-17+ CD4+ ICOS+ CXCR5+ follicular helper T cells. J Immunol 181: 6038–50. 18941193 10.4049/jimmunol.181.9.6038 PMC2753458

[29] Duan W, So T, Mehta AK, Choi H, Croft M (2011) Inducible CD4^+^LAP^+^Foxp3^-^ regulatory T cells suppress allergic inflammation. J Immunol 187: 6499–507. 22079987 10.4049/jimmunol.1101398 PMC3237738

[30] Chen ML, Yan BS, Bando Y, Kuchroo VK, Weiner HL (2008) Latency-associated peptide identifies a novel CD4^+^CD25^+^ regulatory T cell subset with TGF-beta-mediated function and enhanced suppression of experimental autoimmune encephalomyelitis. J Immunol 180: 7327–37. 18490732 10.4049/jimmunol.180.11.7327 PMC2771858

[31] Zhong Y, Wang X, Ji Q, Mao X, Tang H, et al (2012) CD4(+)LAP (+) and CD4 (+)CD25 (+)Foxp3 (+) Regulatory T Cells Induced by Nasal Oxidized Low-Density Lipoprotein Suppress Effector T Cells Response and Attenuate Atherosclerosis in ApoE(-/-) Mice. J Clin Immunol 32: 1104–1. 22552859 10.1007/s10875-012-9699-7

[32] Yasue H, Horio Y, Nakamura N, Fujii H, Imoto N, et al (1986) Induction of coronary artery spasm by acetylcholine in patients with variant angina: possible role of the parasympathetic nervous system in the pathogenesis of coronary artery spasm. Circulation 74: 955–63. 3769179 10.1161/01.cir.74.5.955

[33] Ganta CK, Helwig BG, Blecha F, Ganta RR, Cober R, et al (2006) Hypothermia-enhanced splenic cytokine gene expression is independent of the sympathetic nervous system.Am J Physiol Regul Integr Comp Physiol. 291: R558–65. 10.1152/ajpregu.00846.2005 16469832

[34] Askenasy N, Kaminitz A, Yarkoni S (2008) Mechanisms of T regulatory cell function. Autoimmun Rev 7: 370–5. 18486924 10.1016/j.autrev.2008.03.001

[35] Crispin JC, Martínez A, Alcocer-Varela J (2003) Quantification of regulatory T cells in patients with systemic lupus erythematosus. J Autoimmun 21: 273–6. 14599852 10.1016/s0896-8411(03)00121-5

[36] Viglietta V, Baecher-Allan C, Weiner HL, Hafler DA (2004) Loss of functional suppression by CD4+CD25+ regulatory T cells in patients with multiple sclerosis. J Exp Med 971: 9–19. 10.1084/jem.20031579 PMC2211881 15067033

[37] Kukreja A, Cost G, Marker J, Zhang C, Sun Z, et al (2002) Multiple immuno-regulatory defects in type-1 diabetes. J Clin Invest 109: 131–40. 11781358 10.1172/JCI13605 PMC150819

[38] Tang TT, Ding YJ, Liao YH, Yu X, Xiao H, et al (2010) Defective circulating CD4^+^CD25^+^Foxp3^+^CD127 (low) regulatory T-cells in patients with chronic heart failure. Cell Physiol Biochem 25: 451–8. 20332626 10.1159/000303050

[39] Tang TT, Zhu ZF, Wang J, Zhang WC, Tu X, et al (2011) Impaired thymic export and apoptosis contribute to regulatory T-cell defects in patients with chronic heart failure. PLoS One 6: e24272. 21935395 10.1371/journal.pone.0024272 PMC3174174

[40] Sharabi A, Zinger H, Zborowsky M, Sthoeger ZM, Mozes E (2006) A peptide based on the complementarity-determining region 1 of an autoantibody ameliorates lupus by up-regulating CD4+CD25+ cells and TGF-beta. Proc Natl Acad Sci U S A 103: 8810–5. 16735466 10.1073/pnas.0603201103 PMC1482660

[41] Cheatem D, Ganesh BB, Gangi E, Vasu C, Prabhakar BS (2009) Modulation of dendritic cells using granulocyte-macrophage colony-stimulating factor (GM-CSF) delays type 1 diabetes by enhancing CD4+CD25+ regulatory T cell function. Clin Immunol 131: 260–70. 19171501 10.1016/j.clim.2008.12.001 PMC2701651

[42] Tang TT, Yuan J, Zhu ZF, Zhang WC, Xiao H, et al (2012) Regulatory T cells ameliorate cardiac remodeling after myocardial infarction. Basic Res Cardiol 107: 232. 22189560 10.1007/s00395-011-0232-6

[43] Kohm AP, Carpentier PA, Anger HA, Miller SD (2002) Cutting edge: CD4^+^CD25^+^regulatory T cells suppress antigen-specific autoreactive immune responses and central nervous system inflammation during active experimental autoimmune encephalomyelitis. J Immunol 169: , 4712–4716. 10.4049/jimmunol.169.9.4712 12391178

[44] De Boer OJ, van der Meer JJ, Teeling P, van der Loos CM, van der Wal AC (2007) Low numbers of FOXP3 positive regulatory T cells are present in all developmental stages of human atherosclerotic lesions. PLoS One 2: e779. 17712427 10.1371/journal.pone.0000779 PMC1945014

[45] Han SF, Liu P, Zhang W, Bu L, Shen M, et al (2007) The opposite-direction modulation of CD4^+^CD25^+^ Tregs and T helper 1 cells in acute coronary syndromes. Clin Immunol 124: 90–7. 17512253 10.1016/j.clim.2007.03.546

[46] Zhang WC, Wang J, Shu YW, Tang TT, Zhu ZF, et al (2012) Impaired thymic export and increased apoptosis account for regulatory T cell defects in patients with non-ST segment elevation acute coronary syndrome. Biol Chem 287: 34157–66. 10.1074/jbc.M112.382978 PMC3464524 22872639

[47] Wang R, Zhu J, Dong X, Shi M, Lu C, et al (2012) GARP regulates the bioavailability and activation of TGF-β. Mol Biol Cell. 23: 1129–39. 10.1091/mbc.E11-12-1018 PMC3302739 22278742

[48] Oida T, Weiner HL (2010) TGF-βinduces surface LAP expression on murine CD4 T cells independent of Foxp3 induction. PLoS. One. 5: e15523. 10.1371/journal.pone.0015523 PMC2991360 21124798

[49] Erren M, Reinecke H, Junker R, Fobker M, Schulte H, et al (1999) Systemic inflammatory parameters in patients with atherosclerosis of the coronary and Peripheral arteries. Arterioscler Thromb Vasc Biol 19: 2355–63. 10521364 10.1161/01.atv.19.10.2355

[50] Nakamura-Wakatsuki T, Oyama N, Yamamoto T (2012) Local injection of latency-associated peptide, a linker propeptide specific for active form of transforming growth factor-beta1, inhibits dermal sclerosis in bleomycin-induced murine scleroderma. Exp Dermatol 21: 189–94. 22188586 10.1111/j.1600-0625.2011.01419.x

[51] Boswell S, Sharif S, Alisa A, Pereira SP, Williams R, et al (2011) Induction of latency-associated peptide (transforming growth factor-β (1)) expression on CD4+ T cells reduces Toll-like receptor 4 ligand-induced tumour necrosis factor-α production in a transforming growth factor-β-dependent manner. Immunology 133: 278–287. 21426338 10.1111/j.1365-2567.2011.03425.x PMC3112337

